# Long‐term changes in habitat selection and prey spectrum in a reintroduced Eurasian lynx (*Lynx lynx)* population in Switzerland

**DOI:** 10.1002/ece3.8614

**Published:** 2022-02-22

**Authors:** Daniela Nagl, Urs Breitenmoser, Klaus Hackländer, Andreas Ryser, Fridolin Zimmermann, Sven Signer, Heinrich Haller, Christine Breitenmoser‐Würsten, Kristina Vogt

**Affiliations:** ^1^ Institute of Wildlife Biology and Game Management University of Natural Resources and Life Sciences Vienna Austria; ^2^ KORA Carnivore Ecology and Wildlife Management Muri bei Bern Switzerland; ^3^ Bayerischer Jagdverband e.V Feldkirchen Germany; ^4^ Deutsche Wildtier Stiftung Hamburg Germany; ^5^ 183225 Schweizerischer Nationalpark Zernez Switzerland

**Keywords:** Eurasian lynx, habitat selection, prey spectrum, resource selection function, VHF/GPS telemetry

## Abstract

When wild‐caught Eurasian lynx (*Lynx lynx*) from the Slovak Carpathian Mountains were reintroduced to Central Switzerland in the early 1970s and spread through the north‐western Swiss Alps (NWA), they faced a largely unfamiliar landscape with strongly fragmented forests, high elevations, and intense human land use. For more than 30 years, radio‐collared lynx have been monitored during three different project periods (in the 1980s, 1990s, and 2010s). Our study explored, how lynx over generations have learned to adjust to the alpine environment. We predicted that (1) lynx nowadays select more strongly for open habitats, higher elevations, and steep slopes compared to the early stages of recolonization and that (2) consequently, there were significant changes in the Eurasian lynx’ prey spectrum. To test our predictions, we analyzed telemetry data (VHF, GPS) of 13 adult resident lynx in the NWA over 35 years, using Resource Selection Functions. Furthermore, we compared kills recorded from different individuals inhabiting the same region during three project periods. In general, lynx preferred forested areas, but over the years, they avoided open habitat less. Compared to the early stage of the recolonization, lynx in the most recent project period selected for higher elevations and the proportion of chamois in their prey spectrum surmounted that of roe deer. Potential driving factors for the observed changes could be increasing tolerance to human presence, intraspecific competition, or fitness benefits through exploitation of new resources. Long‐term studies like ours provide important insight into how animals can respond to sudden environmental changes, e.g., in the course of translocations into new areas or anthropogenic alterations of their habitats.

## Graphical abstract

Our study explored, whether reintroduced Eurasian lynx to the north‐western Swiss Alps, over generations have learned to adjust to the Alpine environment. Compared to the early period of recolonization, lynx nowadays selected for higher elevations and avoided open habitats less. The proportion of Alpine chamois in lynx prey spectrum surmounted that of roe deer. Driving factors for the observed changes could be increasing tolerance to human presence, intraspecific competition or fitness benefits through exploitation of new resources.

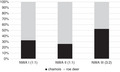

## 1 INTRODUCTION

Large carnivores were almost entirely eradicated from Western and Central Europe by the end of the 19th century. The main reasons for their demise were human persecution, habitat destruction, and loss of their prey base (Breitenmoser, 1998). In recent decades, carnivore species, such as the grey wolf (*Canis lupus*) or the Eurasian lynx (*Lynx lynx*), have successfully re‐established stable or growing populations in many parts of Europe (Chapron et al., 2014). This conservation success has been aided by protective legislation, growing populations of wild ungulates, forest regeneration and—in the case of the Eurasian lynx—by several reintroduction programs (Breitenmoser et al., 2021).

However, with most natural areas in Europe largely destroyed and fragmented by humans due to land use practices, including forestry, agriculture, tourism, and power supply industry, wildlife species nowadays live in human‐modified habitats. In these altered landscapes, the management and conservation of large carnivores are especially challenging due to their large spatial requirements and great potential for conflicts with human activities (Chapron et al., 2014; Schadt et al., 2002). Current threats to large carnivore populations include habitat fragmentation, illegal killing, and loss of genetic diversity in small and isolated populations (LCIE, 2021). For lynx conservation in such human‐modified landscapes, it is essential to understand their space use and habitat choice, in order to predict how anthropogenic changes to landscape are likely to affect lynx habitat suitability and ultimately population viability (Bouyer et al., 2015; Grilo et al., 2019; Penteriani et al., 2018).

The Eurasian lynx is a very widespread species and occurs throughout Europe and large parts of Asia (Breitenmoser et al., 2015). There are six recognized subspecies which show a large variation regarding their morphology, habitat, and prey preferences (Kitchener et al., 2017). In Asia, Eurasian lynx are specialist predators of leporids (Mengüllüoğlu et al., 2018). Throughout large parts of Europe, their main prey is roe deer (*Capreolus capreolus*), with red deer (*Cervus elaphus*), Northern chamois (*Rupicapra rupicapra*), and smaller mammals as alternative prey (Andrén & Liberg, 2015; Chapron et al., 2014; Gervasi et al., 2014; Jobin et al., 2000; Krofel et al., 2011; Odden et al., 2006; Okarma et al., 1997; Podgórski et al., 2008). In the more northern parts of their distribution range, semi‐domestic reindeer (*Rangifer tarandus*) are the main prey, with domestic sheep, leporids, and birds as alternative prey (Mattisson et al., 2011; Sunde et al., 2000; Valdmann et al., 2005). Eurasian lynx are known to occur across a gradient of different habitats, such as mountainous heaths, boreal, mixed and deciduous forests, forest‐steppes, rugged mountainous steppes, or Mediterranean shrublands (Mahdavi et al., 2020; Mengüllüoğlu et al., 2018; Rauset et al., 2013; Schadt et al., 2002).

In Eastern, Central, and Western Europe, continuous forest cover is generally thought to be a prerequisite for the establishment of viable Eurasian lynx populations (Haller, 1992; Müller et al., 2014; Niedziałkowska et al., 2006; Rozylowicz et al., 2010; Schadt et al., 2002; Zimmermann & Breitenmoser, 2002, 2007). Indeed, the Eurasian lynx is often used as a flagship species for the protection of intact and unfragmented forest ecosystems (Niedziałkowska et al., 2006; Noss et al., 1996). However, recent studies have shown that Eurasian lynx can successfully deal with the trade‐off between avoidance of human activities and the preference of areas with high prey densities, which often positively correlate with areas of intensive human land use (Bouyer et al., 2015; Filla et al., 2017; Gehr et al., 2017). Some studies show that Eurasian lynx increased their use of open habitats and areas with high human disturbance at night, while they preferred dense habitat in undisturbed areas for resting during the day (Bouyer et al., 2015; Filla et al., 2017; Gehr et al., 2017), especially where lynx are persecuted (Magg et al., 2016).

Starting from the 1970s, Eurasian lynx have been reintroduced to different forest ecosystems and mountain ranges in Western and Central Europe, such as the Bavarian–Bohemian Forest, the Alps as well as the Jura, Vosges, and Dinaric mountains (Breitenmoser & Breitenmoser‐Würsten, 2008; Drouet‐Hoguet et al., 2021; Fležar et al., 2021; Germain & Schwoerer, 2021; Wölfl et al., 2021). For most reintroductions, wild‐caught Carpathian lynx (*Lynx lynx carpathicus*)—representing the geographically closest remnant population—were used (Breitenmoser et al., 2021). Between 1971 and 1980, lynx captured from the autochthonous population in the Slovak Carpathian Mountains were first released in Switzerland in the Alps and the Jura Mountains (Breitenmoser & Breitenmoser‐Würsten, 2008). Not all of these releases led to a successful establishment of a population nucleus, but nevertheless growing lynx populations have since established in the Swiss and French part of the Jura mountains as well as in the north‐western Swiss Alps (NWA, Breitenmoser & Breitenmoser‐Würsten, 2008). In those parts of the Slovak Carpathians, where lynx occur, forest cover ranges between 60% and 90% (Kubala et al., 2019). Therefore, when lynx were translocated from the Carpathian Mountains to the NWA, they faced a largely unfamiliar environment. Elevations range up to 4273 m a.s.l, the valley bottoms are occupied by human settlements and instead of densely forested slopes, the NWA comprise a high amount of man‐made pastureland in the montane and subalpine zone. Forests cover only about 30% of the area and are strongly fragmented, with timber line lowered by several hundred meters (Vogt et al., 2016).

In a long‐term study area situated in the NWA, research on Eurasian lynx started in the 1980s and since then has been conducted throughout three different project periods: NWA I (1983–1985), NWA II (1997–2001), and NWA III (2011–2017) (Breitenmoser & Haller, 1993; Breitenmoser‐Würsten et al., 2001; Molinari‐Jobin et al., 2007; Vogt et al., 2018). In the early 1980s (NWA I), lynx had only colonized the northern ranges of the Alps with a higher share of forest cover. Haller and Breitenmoser (1986) concluded that the more elevated and less forested landscapes further south did not provide enough suitable habitat for lynx to settle there permanently. In the 1990s (NWA II), however, lynx had also colonized these areas initially considered less suitable and had started using open habitats more and avoiding agricultural land less than expected (Breitenmoser‐Würsten et al., 2001). This raised the question, whether lynx in Switzerland were adjusting their spatial behavior to the new habitat in the cultivated landscape of the NWA and whether the behavioral plasticity of this species, and possibly its adaptive potential, could be higher than originally anticipated.

The semi‐open landscapes of the NWA, with their montane and subalpine altitudinal levels transferred into alpine pastures, imply a higher human presence but also offer valuable additional resources. For example, open habitats above the timber line, as well as steep forested slopes are the preferred habitat of chamois (Schnidrig‐Petrig & Salm, 2009). While the Tatra chamois (*R*.* r*. *tatrica)* is a rare mountain ungulate in the Carpathian Mountains (Anderwald et al., 2020; Corlatti et al., 2022), the Alpine chamois (*R*. *r*. *rupicapra)* is the most abundant ungulate in the NWA (LANAT, 2019).

The aim of our study was to investigate whether Eurasian lynx have adjusted their habitat selection subsequent to their reintroduction. We predicted that (1) lynx nowadays select more strongly for open habitats, higher elevations, and steep slopes compared to the early stages of recolonization (NWA I, NWA II) and that (2) consequently, there were significant changes in the Eurasian lynx’ prey spectrum reflecting the altered habitat use, i.e., a higher predation of Alpine chamois. While behavioral plasticity of habitat selection in mammals has been studied along environmental gradients (Fortin et al., 2008), there are only few studies addressing long‐term changes in habitat use (Ciach & Pęksa, 2019; Fierro‐Calderón & Martin, 2020). Our unique dataset comprising telemetry data over a period of 35 years allows us to investigate changes in spatial behavior over time. Especially for long‐living species like large carnivores, such studies remain rare and can provide important information for future conservation and reintroduction programs (Smith et al., 2017).

## 2 MATERIALS AND METHODS

### 2.1 Study area

**FIGURE 1:**
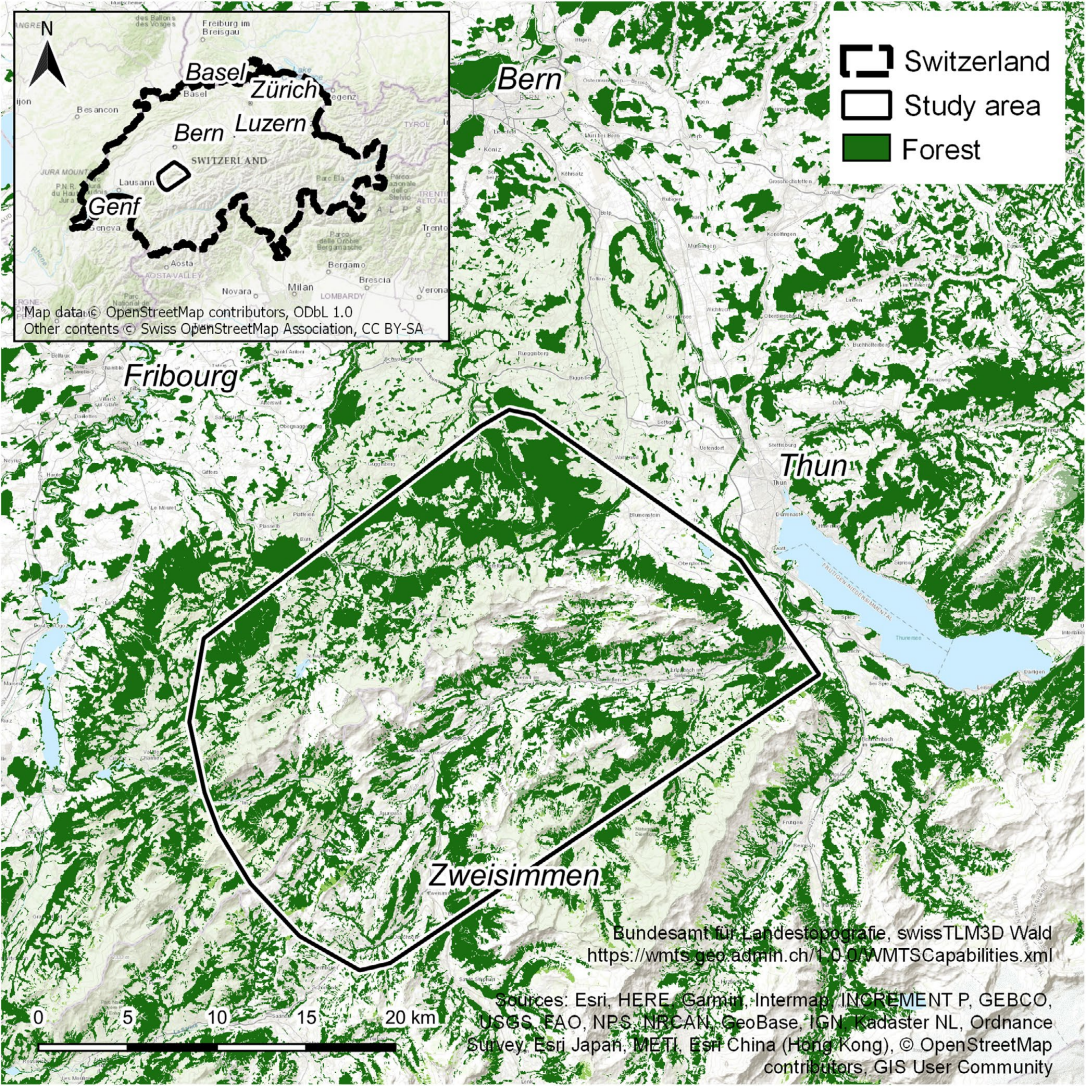
Location of the study site within Switzerland (inset). The black polygon indicates the 95% Minimum Convex Polygon for all telemetry locations of observed lynx (*N* = 13). Green areas represent forest

**FIGURE 2:**
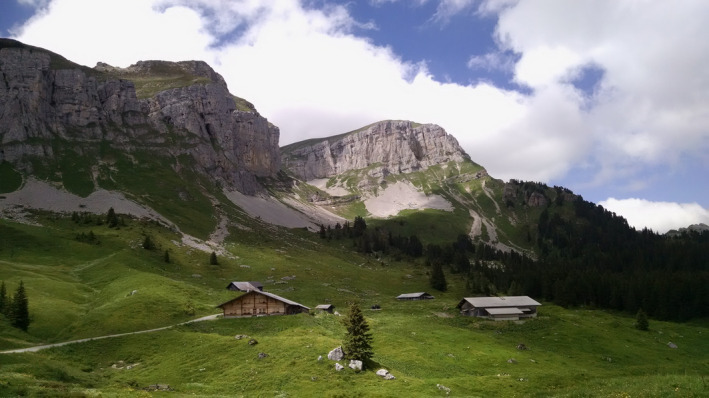
Exemplary picture of open habitat in the north‐western Swiss Alps: Seasonally managed alpine pastures with small forest patches and rock

The study site is mainly situated in the Bernese Oberland, a mountainous area in the NWA in the Canton of Berne (Figure 1). The study area comprises 634 km² and altitude ranges from 600 m to 2230 m a.s.l. The forested area is distributed along the valley sites, strongly fragmented by pastures and human settlements, and covers roughly 40% of the study area. Forests are mainly composed of spruce (*Picea abies*), fir (*Abies alba*), and beech (*Fagus sylvatica*). The vegetation composition ranges from mixed deciduous forest (22%) to (sub)alpine forest communities (78%) (KAWA, 2015). Above the timber line (between 1800 m and 1950 m a.s.l.), habitats are comprised of alpine herbaceous species, krummholz and crag. Hereinafter, when speaking about open habitat, we refer to the upper montane and subalpine zone with seasonally managed alpine pastures, rock, and small forest patches (Figure 2). The most abundant ungulate species in our study area are Alpine chamois and roe deer (LANAT, 2021; Vogt et al., 2019). Red deer occur at low numbers but have not yet been found in lynx prey (von Arx et al., 2017). Yearly censuses of ungulate species are carried out by the cantonal game wardens using direct observations from vantage points for Alpine chamois and spotlight counts along transects for roe deer. Censuses were carried out in late autumn and early spring and census data represents population numbers in spring before reproduction (more detail in Vogt et al., 2019). While there is a potential bias to these census methods (e.g., Meriggi et al., 2008), censuses were carried out by the same observers throughout all three project periods (N. Blatter, pers. Comm.).

### 2.2 Lynx captures and collaring

Lynx were captured between 1983 and 2016 by means of three capture systems. Since the beginning, lynx have been captured either with foot snares set at fresh kills or in large double‐door live traps set on narrow paths (Breitenmoser & Haller, 1993; Vogt et al., 2016). Since 2005, a remotely controlled teleinjection system (Ryser et al., 2005) was additionally used. All lynx were captured following established standard protocols (described in (Ryser et al., 2005; Ryser‐Degiorgis et al., 2002; Vogt et al., 2016) with all permits required by Swiss legislation for capturing, immobilizing, and radio‐tagging lynx.

In the NWA I project, lynx were immobilized either with a blowpipe (box‐traps) or direct intramuscular injection (foot snares) with 0.8–1.5 ml of Ketamine/Xylacine mixture (583 mg Bayer “Rompun” dissolved in 4 ml Parke‐Davis “Ketavet”) (Breitenmoser & Haller, 1993). In later project periods (NWA II and NWA III), lynx were anesthetized with a combination of 0.1–0.15 mg/kg medetomidine hydrochloride (Domitor^®^, Orion Corporation, Espoo, Finland) and 3.2–5.5 mg/kg ketamine hydrochloride (Ketasol^®^, E. Graeub, Berne, Switzerland). As an antagonist, atipamezole hydrochloride (Antisedan^®^, Orion, Corporation, Espoo, Finland) at 0.56–0.77 mg/kg was injected at least 1 hour after the last ketamine injection. This ensures a full metabolization of ketamine and enables interruption of anesthesia in case of complications (Ryser‐Degiorgis et al., 2002).

Animals were fitted with either VHF (~200 g; K. Wagener, Cologne, Germany) or GPS/GSM collars (GPS Plus Mini‐1 C collars, Vectronic Aerospace GmbH, Berlin, Germany; Wild Cell SL/SD GPS‐GSM collars, LoTek wireless, Ontario, Canada), weighing 250–300 g and featuring a break‐off device (seam stitched with 1.2–1.5 mm corrodible annealed wire).

### 2.3 Lynx location data

Data were gathered as part of three research projects (NWA I, 1984–1988; NWA II, 1997–2000; and NWA III, 2011–2017; Table 1) in the north‐western Swiss Alps (projects conducted by KORA; Carnivore Ecology and Wildlife Management, www.kora.ch). VHF telemetry was used during the NWA I and NWA II projects, while lynx observed during the NWA III project were fitted with GPS/GSM collars. This study is based on telemetry data obtained from 13 resident adult lynx (five males, eight females), which were observed for at least 9 months (Table 1). The average duration lynx were monitored was 23 months for VHF, and 17 months for GPS/GSM collar data. The number of locations per individual used for this analysis ranged between 80 and 555 (mean = 274, *SD* = 155).

**TABLE 1:** Sample size overview

Study period	Individual	Number of locations	Observation period (months)	95% Kernel (km^2^)
				
NWA I (1984–1988)	BORA	293 (22)	14	156
	**SEPP**	**259 (25)**	**14**	**267**
	HERA	126 (54)	25	122
	**KOBI**	**83 (41)**	**16**	**160**
NWA II (1997–2000)	KORA	268 (145)	40	137
	**NICO**	**88 (48)**	**14**	**110**
	SABA	412 (221)	47	92
	TANA	80 (29)	12	88
NWA III (2011–2017)	EYWA	479	22	85
	MARI	209	9	112
	**MISO**	**555**	**23**	**108**
	NEVE	374	16	94
	**PIRO**	**339**	**13**	**191**
Total	**13**	**3565**		

Note

Location data were obtained from 13 adult and resident lynx from three different study periods in the north‐western Swiss Alps (NWA). The sample includes five males (in bold; 1324 locations) and eight females (2241 locations). VHF telemetry was used during the NWA I and NWA II study periods. GPS telemetry was used during the NWA III study period. The VHF dataset includes locations of accuracy levels 2 to 4. The number in brackets indicates the share of locations of accuracy 2. The GPS telemetry dataset was reduced to one location per day.

### 2.4 Searching for kill sites

#### 2.4.1 Triangulation of VHF Signal and “homing in”

When lynx were tracked by means of VHF telemetry, five levels of accuracy were distinguished (Breitenmoser‐Würsten et al., 2001). Triangulation was used to determine the lynx’ approximate position and the exact position was affirmed whenever possible by “homing in” on the signal until a level 3 or level 4 accuracy could be reached.

- Level 0: Lynx searched, but not found on that day;
- Level 1: VHF signal heard from one direction, but not localized more precisely;
- Level 2: position located with an accuracy of ±500 m (at least 3 bearings);
- Level 3: position located with an accuracy of ±50 m;
- Level 4: exact position affirmed through direct observation, a kill or tracks.

Lynx individuals were monitored ranging from once per week to several times per day. Lynx usually stay at one place during daytime (Breitenmoser & Haller, 1987) and return to their prey after dusk and during the night (Breitenmoser & Haller, 1993). When a lynx was located at the same spot for a longer time during the evening, it was assumed to have a kill and the surrounding area was searched for prey remains the following day, using a trained dog whenever possible.

#### 2.4.2 GPS location cluster control

With the use of GPS/GSM collars, searching for kill sites became a lot less time consuming. GPS collars were programmed to obtain seven GPS fixes per day, with a higher resolution during twilight and night hours: 01:00, 04:00, 13:00, 17:00, 19:00, 20:00, and 21:00 CET. To locate kill sites, ground‐truthing of GPS location clusters (GLC) was used as described in Vogt et al. (2018).

### 2.5 Statistical analyses

All statistical analyses were performed in R software (version 3.3.3, R Development Core Team, 2015).

#### 2.5.1 Data structure

Since the VHF location dataset was strongly biased toward daylight hours and lynx were often located only once per week to once per day, we restricted the GPS dataset to similar observation times to make it comparable to the field effort invested during the previous VHF telemetry studies. The reduction of the GPS dataset to one fix during daytime between 12:00 and 02:00 CET for all GPS‐collared lynx resulted in 1956 GPS locations (Table 1). The VHF dataset contained 11.2% of localizations of accuracy levels 0 and 1, which were excluded from the analyses, as they lack coordinates. Localizations of accuracy level 2 made up 32.4% and were also excluded, as they do not allow for an accurate assignment to a specific habitat type (accuracy ±500 m). For the habitat analyses, we used localizations of levels 3 and 4 only (1024 locations; 56.4%). In total, 2980 lynx locations (GPS, VHF) were used for the purpose of this study (Table 1).

#### 2.5.2 Calculating home ranges

We estimated home range sizes by generating a 95% fixed kernel with a smoothing factor set to 1000, using the R package *adehabitat* (Calenge, 2006). The smoothing factor was evaluated visually to select the most biologically sensible estimate (Peters et al., 2015). Data points outside the estimated 95% Kernel were considered as outliers in individual range use and excluded from further analyses (Filla et al., 2017).

#### 2.5.3 Modelling habitat use (Resource Selection Function)

Based on proposed scales of habitat selection (Johnson, 1980), we used resource selection functions (RSFs) to assess lynx’ habitat selection within their home ranges (third‐order selection) under a use‐availability design (Manly et al., 2002). RSFs analyze spatial patterns of animal locations obtained from telemetry studies, and have become a widespread method to identify habitat types that are used disproportionately in relation to their availability (Moorcroft & Barnett, 2008). To define resource availability, we generated a set of random ‘available’ locations within home ranges equal to the number of ‘used’ locations obtained from each individual lynx (Peters et al., 2015).

We used a 100 × 100 m grid raster map with 72 basic categories of land use and reclassified the land use categories into three habitat types considered to be relevant for the interests of this study: unsuitable habitat (settlements, water bodies), open habitat (primarily alpine meadows, pastures, and rock), and forest (all kinds of forest, including groups of trees, reforestations, copses, and hedges).

Although land use in Switzerland has changed over the last 35 years, the differences between land cover categories in the study areas across the three study periods were <1% and thus negligible. Hence, analyses were carried out with the most recent available raster map (BFS, BFS‐Arealstatistik 2004/09).

The topographic parameters elevation (m a.s.l.) and slope (degrees) were derived from a digital elevation model (DEM, 25 × 25 m resolution) using the toolbox Spatial Analyst in ArcGIS (ESRI Inc., Redlands, CA, USA). Each ‘used’ or ‘available’ point was attributed with the corresponding reclassified value for land cover (unsuitable habitat, open habitat or forest), elevation, and slope.

We evaluated factors influencing Eurasian lynx’ habitat selection using the function glmer of the R package *lme4* (Bates et al., 2015) to fit generalized linear mixed models (GLMM) by maximum likelihood (Adaptive Gauss‐Hermite Quadrature, nAGQ = 0, family = binomial). Lynx habitat use was the dependent variable (binary response variable: 1 = used, 0 = available). Fixed factors included the continuous variables slope and elevation (standardized). Since ‘habitat’ was a categorical variable, we used ‘forest’ as the reference category. All habitat estimates are in comparison with this reference. To account for changes in habitat use among the three project periods, we included the variable ‘project’ as a factor. The latest project period (NWA III) served as reference because the main focus was on differences in behavior between now and then. The model does not include all possible two‐way interactions, only the ones relevant for hypotheses testing. Individual identity was added as a random effect to our model in order to account for individual preferences and for differences in sample size (Gillies et al., 2006).

Model fit was evaluated by comparing our model to the Null model by means of ΔAIC and by k‐fold cross‐validation as recommended in Boyce et al. (2002). Our Null hypothesis assumed no relationship between project period and lynx habitat choice. Accordingly, to create the Null model, we randomly reassigned the factor levels of the variable ‘project’ to our data and ran the same GLMM as for the main analysis. We performed repeated 5‐fold cross‐validation with 10 repeats and calculated the cross‐validated Spearman‐rank correlations between RSF bin ranks and area‐adjusted frequencies (as in Boyce et al., 2002) using the function kfoldRSF of the R package *IndRSA* (Bastille‐Rousseau & Wittemyer, 2019).

#### 2.5.4 Controlling for methodological bias

Since lynx tracking by means of VHF telemetry can be difficult in mountainous areas (steep and rugged terrain, reflection of signal by rocks) and, therefore, may result in imprecise telemetry locations, the estimated use of certain habitat types by lynx in the early phase of recolonization may have been biased or underestimated in such terrain. For VHF locations with a low accuracy (level 2, ±500 m), the assignment to a specific habitat type may be incorrect. However, if locations in rugged terrain and at higher elevations were more difficult to obtain, leaving out these data could introduce a systematic bias into the VHF dataset. To estimate the expected bias, we conducted a Kruskal–Wallis test for the VHF telemetry data, by comparing elevation and slope at locations of low accuracy (2) to locations of high accuracy (3 and 4). We additionally ran the RSF including level 2 locations and evaluated potential changes in our results (see Appendix A). Model fit of the model including level 2 locations was evaluated in the same way as for the main model,

#### 2.5.5 Analyzing prey spectrum

For comparison of prey spectrum, we only considered ungulate kills (roe deer and Alpine chamois) found by means of VHF telemetry or ground‐truthing of GLCs. Since the main effort for searching for kills in the NWA I and NWA II projects was in winter (November to April), we excluded kills detected during summer months from all datasets for better comparability. Topography and prey community may vary widely within different habitats, so we only considered 10 lynx (five females, five males) inhabiting our study area during different time periods and having at least five kills recorded (ranging from five to 29 kills per individual). Since sex ratio was approximately balanced between project periods, we pooled the data for males and females and conducted a Fisher's exact test for a comparison of prey spectrum between study periods.

## 3 RESULTS

### 3.1 Changes in habitat use within home ranges

**FIGURE 3:**
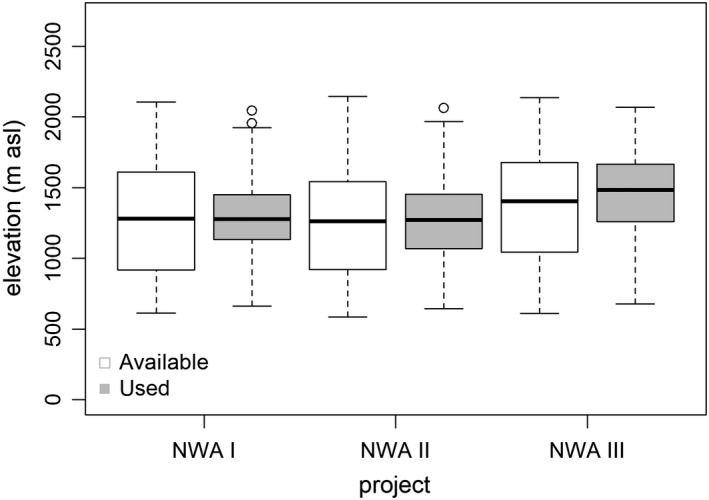
Boxplot showing used (grey boxes) and available (white boxes) elevations within lynx home ranges in the NWA during three different project periods. Each box encompasses the 25th to 75th percentiles, with the median represented by an interior line. Whiskers denote maximum values or, in case of outliers, 1.5 times the interquartile range. Circles denote outliers

**FIGURE 4:**
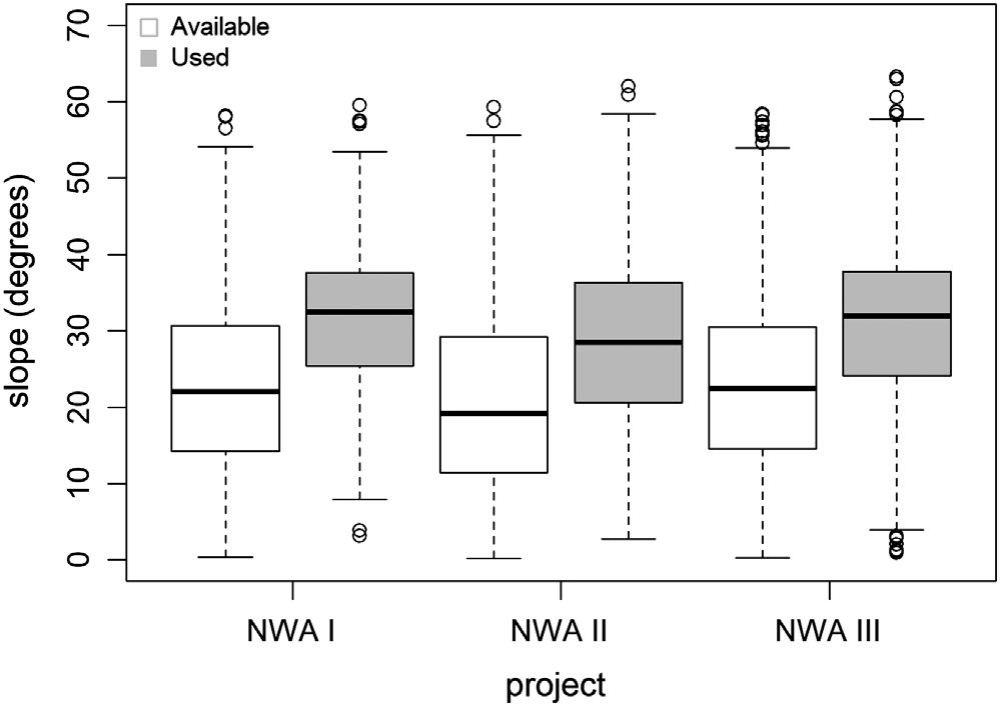
Boxplot showing used (grey boxes) and available (white boxes) slopes within lynx home ranges in the NWA during three different project periods. Each box encompasses the 25th to 75th percentiles, with the median represented by an interior line. Whiskers denote maximum values or, in case of outliers, 1.5 times the interquartile range. Circles denote outliers

During the most recent project period (NWA III), lynx selected more strongly for high elevations and avoided open habitats less than during the earliest project period after reintroduction (NWA I; Table 2, Figure 3). When comparing NWA III to NWA II, there was no significant difference in the selection of open habitats, but lynx of NWA III selected for high elevations more strongly (Table 2, Figure 3). There was no significant difference in selection of slope between project periods (Figure 4). Lynx strongly avoided unsuitable habitat during all three project periods.

**TABLE 2:** Model output of the RSF for lynx habitat selection

Fixed Factors	Estimate	*SE*	*95% CI*		*z*‐value	*p*‐value
			Lower	Upper		
Model Intercept	−2.605	0.195	−2.988	−2.222	−13.334	**<.001**
Open (Habitat)	−1.282	0.080	−1.440	−1.125	−15.998	**<.001**
Unsuitable (Habitat)	−1.255	0.314	−1.871	−0.639	−3.995	**<.001**
Slope	0.057	.004	0.050	0.064	16.164	**<.001**
Elevation	0.120	0.013	0.095	0.145	9.370	**<.001**
NWA I	1.178	0.412	0.371	1.985	2.861	**.004**
NWA II	2.413	0.341	1.744	3.082	7.066	**<.001**
Slope: NWA I	0.009	0.008	−0.007	0.025	1.121	.262
Slope: NWA II	−0.012	0.008	−0.026	0.003	−1.562	.118
Elevation: NWA I	−0.102	0.029	−0.159	−0.045	−3.493	**<.001**
Elevation: NWA II	−0.151	0.026	−0.202	−0.100	−5.772	**<.001**
Open: NWA I	−0.410	0.186	−0.774	−0.045	−2.202	**.028**
Unsuitable: NWA I	−0.348	0.752	−1.822	1.126	−0.463	.643
Open: NWA II	0.090	0.162	−0.229	0.408	0.552	.581
Unsuitable: NWA II	0.040	0.576	−1.090	1.169	0.069	.945

Note

Positive parameter estimates correspond to preference, whereas negative coefficients correspond to avoidance. ‘Forest’ was the reference category for habitat types. Study period NWA III was the reference for the variable ‘project’. The analysis was conducted on 2980 locations and an equal number of random points. Lynx identity (estimated variance component = 0.007, *SD* = 0.084) was included as random effect. *SE* = Standard Error, *CI* = Confidence Interval. AIC = 7009.

Bold values indicate *p*‐values < .05.

The mean elevation of realized lynx locations during NWA I was 1293 m (*SD* ±215), during NWA II 1268 m (*SD* ±270), and during NWA III 1452 m (*SD* ±268). The highest day location was located at 2068 m (NWA III), the lowest at 645 m (NWA II). In the NWA I study, lynx locations were located at an average slope of 31.6° (±9 *SD*), similar to the NWA III study with 31.1° (*SD* ±11). In the NWA II study, the average used slope was 28.5° (*SD* ±11).

### 3.2 Evaluating potential methodological bias

Locations of accuracy 2 were situated on steeper slopes (Kruskal–Wallis test, *χ*² = 14.262, *df* = 1, *p* =< .001) and at higher elevations (Kruskal–Wallis test, *χ*² = 40.697, *df* = 1, *p* =< .001) than locations of higher accuracy (levels 3 and 4).

The estimated model coefficients for each variable in the model output of the RSFs were similar with and without VHF locations of accuracy 2 (cf. Table 2, Appendix A). Although the differences between the study periods were a little less pronounced when including locations of accuracy 2, the results were still significant (Appendix A).

### 3.3 Model fit

Our model performed better than the Null model (ΔAIC: 65). Also, the model including locations with an accuracy of level 2 (Appendix A) performed better than the corresponding Null model (ΔAIC: 61). The model coefficients for the Null models are shown in Appendix B. The result of the repeated 5‐fold cross‐validation indicated a good model fit with strong cross‐validated Spearman‐rank correlations (*r*_s_) between RSF bin ranks and area‐adjusted frequencies (average *r*_s_ = .992; Appendix C). The performance of the model including locations of accuracy level 2 was comparable (Appendix C).

### 3.4 Changes in prey spectrum

**FIGURE 5:**
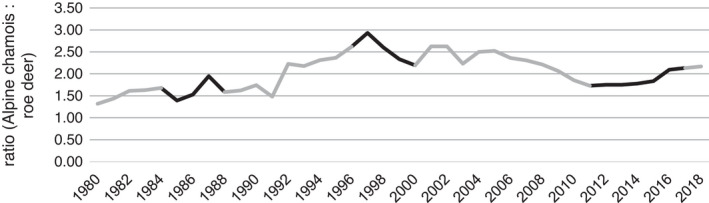
Yearly changes in the ratio of abundance of the two main prey species (Alpine chamois: roe deer) of Eurasian lynx in the study area between 1980 and 2018. The project periods are highlighted in black: NWA I (1984–1988), NWA II (1997–2000), and NWA III (2011–2017). (Data source: yearly census data from the study area; hunting inspectorate of the canton of Bern, LANAT)

**FIGURE 6:**
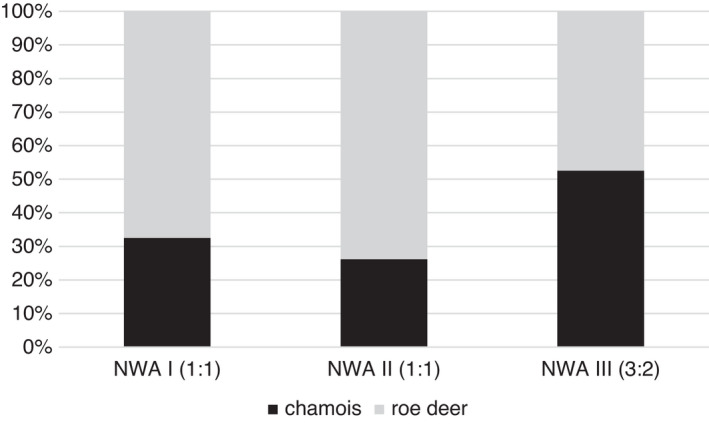
Prey items (*N* = 202) collected during winter (November to April) of radio‐collared lynx (*N* = 11) inhabiting the same region at different times. Numbers in brackets indicate sex ratio of observed lynx for each project period (female: male)

The ratio of abundance between Alpine chamois and roe deer in our study area increased from period NWA I (Ø 1.63) to NWA III (Ø 2.51) by 54% and declined from NWA II to NWA III (Ø 1.87) by 25% (Figure 5). The lynx inhabiting the same region over different time periods showed a significant difference in prey spectrum (Fisher's exact test, *p* = 0.002). The proportion of Alpine chamois increased from 33% in NWA I to 53% of the total prey spectrum in NWA III (Figure 6). The proportion of roe deer decreased over the years from 68% to 47%.

## 4 DISCUSSION

When reintroducing species to environments where they have been extinct, it is essential to evaluate the current suitability of habitat in the destination area and to choose individuals for translocations from the right source populations (IUCN/SSC, 2013). If founder individuals originate from environments markedly different to the destination area, they may be poorly adapted (IUCN/SSC, 2013). When wild‐caught lynx from the rather continuous forests of the Slovak Carpathian Mountains were translocated to the Swiss Alps in the 1970s, they faced a landscape strongly fragmented by human settlements, pastoralism, and a much broader altitudinal range. Our study revealed that reintroduced lynx have adjusted their habitat selection to their new environment during the three decades since reintroduction. Over time, lynx selected more strongly for higher elevations and open areas. Consequently, in the latest generation, Alpine chamois became the main prey species in lynx prey spectrum, displacing roe deer.

### 4.1 Habitat selection

In parts of their distribution range, Eurasian lynx are inhabiting grasslands, Mediterranean shrublands, or alpine tundra (Linnell et al., 2021; Mahdavi et al., 2020; Mengüllüoğlu et al., 2018; Rauset et al., 2013). In Eastern, Central, and Western Europe, however, the Eurasian lynx is often used as a flagship species for the conservation of forest habitats (Niedziałkowska et al., 2006; Noss et al., 1996). Also, in our study, lynx preferred forest in all three project periods, which is in line with previous investigations (Basille et al., 2009; Breitenmoser‐Würsten et al., 2001; Filla et al., 2017; Haller, 1992; Rozylowicz et al., 2010; Zimmermann, 2004; Zimmermann & Breitenmoser, 2002, 2007). However, we observed a weaker avoidance of open areas in more recent project periods (NWA II, NWA III) compared to the earliest project period few years after recolonization (NWA I). This suggests that lynx started using the open habitats of the montane and subalpine zone more frequently already in the 1990s (Breitenmoser‐Würsten et al., 2001) compared to the 1980s, where observed lynx were located primarily in closed forest (Breitenmoser & Haller, 1993; Haller & Breitenmoser, 1986). The lynx observed by Breitenmoser and Haller (1993) were likely the very first generations of offspring raised by reintroduced lynx, i.e., their mothers were translocated from the Carpathians. This suggests that the early generations of lynx born in Switzerland had not yet experienced social transmission of adjusted habitat use.

Overall, open habitats made up more than 50% of the available habitat in all home ranges of observed lynx (except for one individual). To our knowledge, such a high share of open habitat has not yet been documented for lynx home ranges in Central Europe (e.gNiedziałkowska et al., 2006; Odden et al., 2006) and this finding is important to consider for future habitat suitability and connectivity analyses. In our study, lynx telemetry locations occurring in open terrain increased from 17% (NWA I) to 33% (NWA III) over time, whereas locations occurring in forested areas declined significantly from 82% to 66% (Appendix D, Fisher's exact test, *p* = .031). Fragmented forests and areas with intensive human land use are preferred habitat for roe deer (Lorenzini et al., 2022, in press) and Eurasian lynx face a trade‐off between avoiding human disturbance and selecting areas with high prey abundance (Basille et al., 2009; Bouyer et al., 2015; Filla et al., 2017; Gehr et al., 2017). Two recent studies on Eurasian lynx habitat selection have found that lynx solved this trade‐off by avoiding open habitats less during twilight and even by selecting meadows at night when human activity was low (Filla et al., 2017; Gehr et al., 2017). With most telemetry data in our study obtained during daylight hours, we can conclude that lynx also became less avoidant of open habitats during daytime, when they were mainly resting and not hunting.

All observed lynx selected for steeper terrain than available on average. However, contrary to our predictions, there was no significant difference in selection for slope over time. In the most recent project period (NWA III), lynx selected for higher elevations more strongly than in the earlier period of reintroduction. In human‐dominated landscapes, steep slopes and high elevation often correlate with lower levels of human disturbance (Basille et al., 2009; Zimmermann & Breitenmoser, 2002). Large carnivores incur higher energetic costs for movement in steeper terrain and avoid steep slopes in undisturbed areas. In the presence of humans, however, they are known to choose energetically suboptimal movement strategies and relax their avoidance of slope (Nickel et al., 2021; Nisi et al., 2021). Avoidance of humans was a likely driver of the selection for steep slopes across all project periods in our study area. However, it does not fully explain the stronger selection of high elevations in the most recent project period.

Intra‐guild or intra‐specific competition is another known factor influencing habitat selection in carnivores (O'Neil et al., 2020; Pereira et al., 2012; St‐Pierre et al., 2006). Habitat selection can be density dependent (Boyce et al., 2002; O'Neil et al., 2020). Increasing population densities can drive competitively inferior animals into habitats they would not normally prefer and which may or may not prove suboptimal for their reproductive success (López‐Bao et al., 2011; O'Neil et al., 2020; Svanbäck & Bolnick, 2007). When the lynx population in our study area increased and suitable habitat became saturated (Breitenmoser‐Würsten et al., 2001; Chapron et al., 2014; Kunz et al., 2017; Zimmermann et al., 2012, 2016), lynx may have been forced to better utilize their home ranges vertically.

Another driving factor for habitat selection in large carnivores is prey availability (Bouyer et al., 2015; Cristescu et al., 2019; Davidson et al., 2012; Oakleaf et al., 2006; Roder et al., 2020; Soyumert et al., 2019). Roe deer density has been shown to have a positive influence on lynx occurrence (Bouyer et al., 2015; Müller et al., 2014). Accordingly, Basille et al. (2009) linked the avoidance of alpine areas and higher elevations shown by Eurasian lynx in Southern Norway to the lack of a suitable main prey species in these habitats. In our study area, higher elevations comprise Alpine pastures inhabited by suitable prey species like Alpine chamois, Alpine marmot *Marmota marmota*, and mountain hare *Lepus timidus* (Jobin et al., 2000; Vogt et al., 2018. Hence, the observed vertical home‐range expansion by lynx could have resulted in better exploitation of additional resources (see Figure 6 on prey spectrum), which may in turn have implied fitness benefits through higher food intake (Holekamp & Dloniak, 2010).

### 4.2 Methodological bias

The analysis of long‐term datasets is often characterized by changes in animal tracking technology (Land et al., 2008). This is also the case in our study, where VHF telemetry was superseded by GPS telemetry in the most recent project period. With GPS telemetry, the physical presence of an observer in the field is no longer required to obtain a localization, alleviating potential biases of terrain accessibility. Indeed, in our VHF dataset, locations of lower accuracy were on average in steeper and higher terrain than locations of high accuracy, corroborating the fact that VHF telemetry in steep and inaccessible terrain may result in less accurate data. Potentially, the inability to get close enough to a lynx in inaccessible terrain could also result in failed localization attempts. However, localizations of level 0 (signal not found) or 1 (VHF signal heard only from one direction) only made up 11.2% of the VHF dataset we worked with and their inclusion in the models probably would not have changed the results substantially. Moreover, localizations of level 1 typically did not represent the failure of achieving a more accurate localization for a specific target lynx due to terrain inaccessibility, but were rather taken as complementary information, when the signal of a non‐target lynx was heard during localization of a target lynx. When accounting for a potential methodological bias by including locations of accuracy level 2 in our model, the observed effects were slightly less pronounced but still significant and the direction of the observed effects did not change (Appendix A). We thereby conclude that while lynx in the past could have used open habitat at high elevations more frequently than assumed, the observed changes in habitat selection represent a biological effect and not merely a methodological bias.

### 4.3 Prey spectrum

The range of the Northern chamois in Slovakia is restricted to the Tatra mountains (Anderwald et al., 2020; Corlatti et al., 2022, in press). While the exact origin of the first Carpathian lynx translocated to Switzerland is not known for all individuals, those individuals with known origin all came from Slovakia and none came from the Tatra mountains (U. Breitenmoser, pers. comm.). Alpine chamois were, therefore, likely a novel prey item to the reintroduced lynx in Switzerland. The fact that chamois were already part of the lynx’ prey spectrum in the first project period after reintroduction (NWA I) suggests that Eurasian lynx, as well as other large predators, may show individual behavioral plasticity in their prey choice (Holekamp & Dloniak, 2010; Oriol‐Cotterill et al., 2015). Subsequently, lynx have increased the use of Alpine chamois and it became the main prey species during the most recent project period (NWA III). Thus, space use patterns of lynx in our study area may be influenced less by roe deer availability and more by chamois availability than previously assumed (Gehr et al., 2017).

Alpine chamois are more common than roe deer in our study area (LANAT, 2021; Vogt et al., 2019). The relative abundance of chamois compared to roe deer increased in the study area during the 1990s and dropped toward NWA III (cf. Figure 5: ratio chamois/roe deer: NWA I: Ø 1.63, NWA II: Ø 2.51, NWA III: Ø 1.87). From the relative abundance of the two main prey species, we could have expected the highest share of Alpine chamois in lynx diet in the NWA II project period. However, this was not the case and a significant increase of Alpine chamois in lynx diet became evident only in the last project period. Nevertheless, the observed shift toward chamois as main prey is consistent with the observed changes in habitat use over time. This suggests that the lynx’ increased use of open habitats and higher elevations may have led to higher encounter probabilities with Alpine chamois and subsequently to changes in prey spectrum. While there is considerable variation in diet across the Eurasian lynx’ whole distribution range (e.g., in northern and south‐eastern habitats) (Linnell et al., 2021; Mengüllüoğlu et al., 2018), the replacement of roe deer as main prey species (even in areas with low roe deer densities) has rarely been reported in previous studies from Western, Central, and Eastern Europe (Haller, 1992; Moa et al., 2006; Molinari‐Jobin et al., 2007; Nilsen et al., 2009; Odden et al., 2006; Podgórski et al., 2008).

## 5 CONCLUSION

Our study shows that reintroduced Carpathian lynx were able to adapt their habitat selection and diet to the new environment of the NWA. Compared to earlier periods after their reintroduction, lynx today increased their selection of higher elevations and open areas and changed their main prey species from roe deer to Alpine chamois. Potential drivers for the observed changes could be increased tolerance toward human presence (Basille et al., 2009; Bouyer et al., 2015; Filla et al., 2017; Gehr et al., 2017), intraspecific competition (Boyce et al., 2002; O'Neil et al., 2020), or fitness benefits from exploitation of new resources. The observed behavioral adjustments seemed to take several generations to come into effect, either through social transmission or adaptation. Our findings demonstrate that Eurasian lynx can be conserved in human‐modified landscapes with fragmented forests and a high proportion of open habitats. Long‐term studies on changes in habitat use are still rare and provide important insight into how animals can respond to abrupt environmental changes, such as translocations into new areas or anthropogenic alterations of their habitats.

## CONFLICT OF INTEREST

None declared.

## AUTHOR CONTRIBUTION

**Daniela Nagl:** Formal analysis (lead); Investigation (supporting); Methodology (supporting); Writing – original draft (lead); Writing – review & editing (lead). **Urs Breitenmoser:** Conceptualization (lead); Investigation (lead); Writing – review & editing (equal). **Klaus Hackländer:** Writing – review & editing (equal). **Andreas Ryser:** Investigation (lead); Writing – review & editing (supporting). **Fridolin Zimmermann:** Investigation (lead); Writing – review & editing (equal). **Sven Signer:** Investigation (equal); Writing – review & editing (supporting). **Heinrich Haller:** Investigation (lead); Writing – review & editing (equal). **Christine Breitenmoser‐Würsten:** Funding acquisition (lead); Investigation (equal). **Kristina Vogt:** Formal analysis (lead); Investigation (lead); Methodology (lead); Writing – original draft (supporting); Writing – review & editing (lead).

## Data Availability

Data are available from the Dryad Digital Repository: https://doi.org/10.5061/dryad.sf7m0cg7v (Vogt et al., 2022).

## APPENDIX A

Model output of the RSF for lynx habitat selection including VHF localizations of low accuracy (level 2). Positive parameter estimates correspond to preference, whereas negative coefficients correspond to avoidance. ‘Forest’ was the reference category for habitat types. Study period NWA III was the reference category for variable ‘project’. The analysis was conducted on 3565 locations and an equal number of random points. Lynx identity (estimated variance component = 0.016, *SD* = 0.126) was included as random effect. *SE* = Standard Error, *CI* = Confidence Interval. AIC = 8346

**Table:**

Fixed Factors	Estimate	*SE*	95% CI		*z*‐value	*p‐value*
			Lower	Upper		
Model Intercept	−2.596	0.201	−2.989	−2.202	−12.934	**<.001**
Open (Habitat)	−1.281	0.080	−1.439	−1.124	−15.959	**<.001**
Unsuitable (Habitat)	−1.249	0.314	−1.865	−0.633	−3.973	**<.001**
Slope	0.057	0.004	0.050	0.064	16.180	**<.001**
Elevation	0.119	0.013	0.093	0.144	9.178	**<.001**
NWA I	1.166	0.410	0.363	1.970	2.844	.**004**
NWA II	1.968	0.297	1.386	2.550	6.628	**<.001**
Slope: NWA I	0.008	0.008	−0.008	0.023	0.963	.336
Slope: NWA II	−0.005	0.006	−0.016	0.007	−0.799	.424
Elevation: NWA I	−0.096	0.028	−0.151	−0.040	−3.389	.**001**
Elevation: NWA II	−0.129	0.021	−0.170	−0.087	−6.028	**<.001**
Open: NWA I	−0.414	0.179	−0.765	−0.064	−2.318	.**020**
Unsuitable: NWA I	−0.435	0.747	−1.899	1.029	−0.582	.560
Open: NWA II	−0.171	0.720	−0.259	0.253	−0.237	.980
Unsuitable: NWA II	−2.462	0.568	−1.211	0.673	−13.130	.575

Note

Bold values indicate *p*‐values < .05.

## APPENDIX B

Model coefficients of the Null models for lynx habitat selection with and without VHF localizations of low accuracy (level 2). Factor levels of the variable ‘project’ were randomly assigned to the dataset. ‘Forest’ was the reference category for habitat types. Study period NWA III was the reference category for variable ‘project’. Lynx identity was included as random effect (without level 2 locations: estimated variance component = 0.024, *SD* = 0.145; with level 2 locations: estimated variance component = 0.021, *SD* = 0.144). *SE* = Standard Error, *CI* = Confidence Interval

**Table:**

Fixed Factors	Estimate	*SE*	*95% CI*		*z*‐value	*p‐value*
			Lower	Upper		
Null model without level 2 locations (5960 observations); AIC = 7074						
Model Intercept	−1.795	0.178	−2.145	−1.445	−10.066	**<.001**
Open (Habitat)	−1.297	0.079	−1.451	−1.143	−16.468	**<.001**
Unsuitable (Habitat)	−1.128	0.285	−1.687	−0.569	−3.956	**<.001**
Slope	0.054	0.003	0.047	0.061	15.491	**<.001**
Elevation	0.075	0.012	0.050	0.099	6.082	**<.001**
NWA I	−0.195	0.382	−0.944	0.553	−0.511	.609
NWA II	0.143	0.363	−0.569	0.854	0.393	.695
Slope: NWA I	0.007	0.008	−0.008	0.022	0.862	.389
Slope: NWA II	0.002	0.007	−0.012	0.017	0.334	.738
Elevation: NWA I	−0.007	0.027	−0.059	0.045	−0.257	.797
Elevation: NWA II	−0.020	0.026	−0.071	0.030	−0.787	.431
Open: NWA I	0.129	0.173	−0.209	0.468	0.751	.453
Unsuitable: NWA I	−0.012	0.718	−1.419	1.396	−0.016	.987
Open: NWA II	−0.026	0.169	−0.357	0.306	−0.151	.880
Unsuitable: NWA II	−1.076	0.813	−2.668	0.517	−1.323	.186
Null model with level 2 locations (7130 observations); AIC = 8407						
Model Intercept	−1.915	0.180	−2.268	−1.562	−10.625	**<.001**
Open (Habitat)	−1.271	0.079	−1.425	−1.116	−16.117	**<.001**
Unsuitable (Habitat)	−1.475	0.329	−2.120	−0.831	−4.486	**<.001**
Slope	0.058	0.003	0.051	0.065	16.707	**<.001**
Elevation	0.071	0.012	0.046	0.095	5.698	**<.001**
NWA I	0.394	0.361	−0.314	1.102	1.091	.275
NWA II	0.391	0.283	−0.164	0.946	1.381	.167
Slope: NWA I	−0.002	0.007	−0.016	0.013	−0.212	.832
Slope: NWA II	−0.007	0.006	−0.018	0.004	−1.261	.207
Elevation: NWA I	−0.023	0.026	−0.074	0.028	−0.890	.373
Elevation: NWA II	−0.010	0.020	−0.049	0.029	−0.508	.612
Open: NWA I	−0.006	0.168	−0.335	0.323	−0.036	.971
Unsuitable: NWA I	0.314	0.591	−0.845	1.473	0.531	.595
Open: NWA II	−0.114	0.132	−0.372	0.145	−0.862	.389
Unsuitable: NWA II	0.029	0.509	−0.969	1.028	0.058	.954

Note

Bold values indicate *p*‐values < .05.

## APPENDIX C

Cross‐validated Spearman‐rank correlations (*r*_s_) between RSF bin ranks and area‐adjusted frequencies for individual and average model sets (as in Boyce et al., 2002)

**Table:**

Without locations level 2		With locations level 2	
Model set	*r*_s_	Model set	*r*_s_
1	.988	1	1.000
2	.988	2	.997
3	.997	3	1.000
4	.976	4	.997
5	.988	5	1.000
6	1.000	6	.988
7	1.000	7	1.000
8	1.000	8	1.000
9	.988	9	1.000
10	.997	10	.997
Average	.992	Average	.998

## APPENDIX D

Proportion of telemetry locations of lynx in forest and open habitat during three study periods: NWA I (1983–1988), NWA II (1997–2001), and NWA III (2011–2017). The proportion of locations in open habitat increased significantly between study periods (Fisher's exact test, *p* = .031)
